# Stroke rate–stroke length dynamics in elite freestyle swimming: application of kernel density estimation

**DOI:** 10.3389/fspor.2025.1656633

**Published:** 2025-10-08

**Authors:** Craig A. Staunton, Jesús J. Ruiz-Navarro, Dennis-Peter Born

**Affiliations:** ^1^ Department of Environmental and Bioscience, School of Business, Innovation and Sustainability, Halmstad University, Halmstad, Sweden; ^2^ Aquatics Lab, Department of Physical Education and Sports, Faculty of Sport Sciences, University of Granada, Granada, Spain; ^3^ Swiss Development Hub for Strength and Conditioning in Swimming, Swiss Aquatics—National Swimming Federation, Worblaufen, Switzerland; ^4^ Department for Elite Sport, Swiss Federal Institute of Sport Magglingen, Magglingen, Switzerland

**Keywords:** biomechanical analysis, elite athletes, kernel density estimation, pacing strategies, performance visualization

## Abstract

**Objectives:**

To analyse stroke rate (SR) and stroke length (SL) combinations among elite swimmers to better understand stroke strategies across all race distances of freestyle events.

**Design:**

We analysed SR and SL data from 324 male and female swimmers competing in all individual freestyle events (50 m to 1,500 m) at the 2019 European Short-Course Championships using video-based kinematic analysis.

**Methods:**

Two-dimensional kernel density estimation (2D KDE) was applied to visualise SR–SL combinations. Spearman correlations quantified relationships between stroke parameters and speed by sex and race distance.

**Results:**

In the 50 m sprint, SL showed the strongest positive correlation with speed (men: *ρ* = 0.57; women: *ρ* = 0.50), while SR correlations were trivial. As race distance increased, SR correlations with speed strengthened, reaching moderate levels in long-distance events (men's 1,500 m: *ρ* = 0.37; women's 800 m: *ρ* = 0.45), whereas SL correlations weakened. The 2D KDE heatmaps revealed an inverse SR–SL relationship, with medallists often employing stroke strategies distinct from finalists and the broader field. Gold medallists in sprint events favoured above-average SR without compromising SL, while in middle- and long-distance races, a shift toward higher SR and reduced SL was observed, particularly among women.

**Conclusions:**

These findings highlight the complexity and individuality of stroke mechanics at elite levels and suggest that superior conditioning and technique enable medallists to sustain elevated SR without compromising SL. The application of 2D KDE provides a novel, intuitive method to capture nuanced biomechanical strategies, offering valuable insights for coaching and performance optimisation.

## Introduction

In competitive swimming, clean swimming speed—that is, the swimmer's average speed during the stroking phases excluding the effects of starts, turns, and push-offs—is the product of stroke rate (SR) and stroke length (SL). SR refers to the number of complete cycles of 1 arm in a given unit of time, and SL here refers to the distance the swimmer moves forward per stroke (1). By contrast, race speed (or overall speed) is typically calculated from the ratio of the total race distance to the total race time, thereby incorporating the influence of starts, turns, and underwater phases. SR has been linked to neuromuscular power and energy capacities (2), while SL has been related to force production and the ability to effectively apply that force in the water (3). Both are interdependent variables that swimmers must optimise to achieve peak performance (4), rather than compensating the acute increase of one with a decrease of another one. While this relationship appears straightforward, the physiological, biomechanical, and tactical nuances involved in achieving optimal combinations of SR and SL are complex and highly individual (5–7).

A growing body of literature has examined how changes in SR and SL influence swimming speed across different strokes and race distances (5, 7–9). Typically, SR is seen as the primary contributor to higher swim velocities, particularly in sprint events (4, 10). However, research also suggests that this emphasis may oversimplify the dynamic relationship between SR and SL, especially across varying levels of fatigue, technique efficiency, and pacing strategies (11). Notably, some elite swimmers maintain longer SL even at high velocities with shorter SR, challenging the notion that simply increasing stroke rate is the most effective strategy (10, 12).

Performance analysis in sport, particularly race analyses in swimming, often involves identifying the movement patterns adopted by top performers [e.g., (13–15)], under the assumption that their strategies represent near-optimal solutions within real-world constraints (16). This concept aligns with the Aristotelian principle of *telos*, which suggests that the highest expression of an activity reveals its most effective or purposeful form. In this context, the stroke mechanics of world championship medallists may offer valuable empirical insights into high-performance swimming.

Kernel density estimation (KDE) is a statistical technique used to estimate the probability density of bivariate data. In sports science, KDE has been increasingly applied to visualise distributions of technical and tactical behaviours. For example, recent studies have used two-dimensional (2D) KDE to map relationships between internal and external training demands in elite speed skaters (17) and biathletes (18). Given that SR and SL are inherently bivariate and interdependent, 2D KDE is particularly well-suited to visualising how these two variables interact in swimming. Despite this, to date, 2D KDE has not been applied to visualise SR and SL combinations in swimming—representing a novel approach to performance analysis in the sport. By generating heat maps that identify the most densely populated zones of SR–SL combinations, 2D KDE plots might provide an intuitive visual representation of the stroke strategies adopted by world-class swimmers. These visualisations might complement traditional performance analysis techniques by revealing both common and divergent biomechanical patterns across sexes and race distances.

As such, the present study analysed stroke mechanics from elite-level freestyle events and used 2D KDE to visualise the combinations of SR and SL. The aims of the study were to: (1) Identify the most common SR–SL combinations among finalists and medallists; (2) Examine how these combinations vary by sex and race distance; and (3) Discuss the implications of these patterns for coaching, athlete development, and biomechanical modelling in swimming. This study was designed as an exploratory, retrospective analysis without *a priori* hypotheses, aiming instead to characterise SR–SL profiles among elite swimmers and identify patterns associated with medal-winning performances.

## Methods

### Participants

Participants included all swimmers for every individual freestyle race of both the women's (*N* = 154; 50 m: Race Starts = 77; 100 m: Race Starts = 85; 200 m: Race Starts = 56; 400 m: Race Starts = 51; 800 m: Race Starts = 37) and men's (*N* = 170; 50 m: Race Starts = 93; 100 m: Race Starts = 101; 200 m: Race Starts = 64; 400 m: Race Starts = 52; 1,500 m: Race Starts = 30) events at the 2019 European Short-Course Swimming Championships in Glasgow, Scotland. All participants competing at European-championship level are classified as either tier 4 or tier 5 level athletes according to the participant classification framework (19). All swimmers that participate at events hosted by the European Swimming Association LEN (Ligue Européenne de Natation) agree to be video monitored for television broadcasting and race analysis of the participating nations. The study was pre-approved by the leading institution's internal review board (registration number: 098-LSP-191119) and was in accordance with the latest version of the code of conduct of the World Medical Association for studies involving human subjects (Helsinki Declaration).

### Data collection

A retrospective analysis was performed from performance data obtained from the 2019 European Short-Course Swimming Championships. To extract SR and SL data from the free-swimming portions of each lap using video analysis, a twelve-camera system (Spiideo, Malmö, Sweden) was employed to monitor all races. Among these cameras, ten focused on individual swimmers, each capturing one of the ten lanes (V59 PTZ, Axis Communications AB, Lund, Sweden). Additionally, two cameras with a fixed view were placed at a 90° angle to the swimming lanes, one at the 5 m mark and the other at the 20 m mark, to oversee the start and turn sections for all lanes. Video footage was captured at a sampling rate of 50 Hz. The video footages were manually post processed by a single assessor (Kinovea 0.9.1; Joan Charmant & Contrib., https://kinovea.org/). Another expert race analyst analysed five percent of the race in duplicate to determine the inter-rater reliability, which has previously been reported with an intra-class correlation coefficient of 0.98 ± 0.04 (20–22). Race times were provided by the official timekeeper of the championship (Microplus Informatica, Marene CN, Italy).

### Data analyses

For each lap, SR and SL were measured during a 10-metre middle section of free swimming—specifically from the 10 m to the 20 m mark, except for the first lap where only the 15–20 m section was used. These zones were selected to minimise the effects of the wall approach and push-offs during turns (23). Using frame-by-frame video analysis, the start and end of one full stroke cycle within this section were identified to determine the stroke time. SR (in strokes per minute) was calculated by dividing 60 by the stroke time. To determine SL (in metres), the stroke time was multiplied by the swimmer's speed over the particular section. Swimming speed was calculated as section distance (in metres) divided by time to swim that section (measured in seconds determined from the position of the top of the head at the beginning to the end of the free-swimming section).

### Statistical analyses

The normality of data was checked using Kolmogorov–Smirnov Tests and visual inspection of histograms and Q-Q plots. In most cases, the SR or SL were not normally distributed. As such, Spearman rank correlation coefficients (*ρ*) were computed to determine the relationships between both SR and SL and swimming speed for all races distances and both sexes. The strength of relationships was interpreted according to Hopkins et al. (24): trivial (<0.1), small (0.1–0.3), moderate (0.3–0.5), large (0.5–0.7), very large (0.7–0.9) and nearly perfect (>0.9).

KDE analyses were performed in MATLAB (Version R2024a, The MathWorks Inc) using the “*ksdensity*” function, applying a kernel bandwidth as a smoothing parameter (25, 26). Two-dimensional (2D) KDE heatmaps were generated to visualise the coupling of SR and SL for each lap of the particular race (i.e., each swimmer's adopted combination of SR and SL). Warmer colours in the heatmaps (i.e., yellow and red) indicate high density at that particular combination of SR and SL, meaning that particular combination occurred frequently. Conversely, cooler colours (i.e., blue) represent lower density regions, meaning that particular combination occurred infrequently. In addition, the particular combinations of SR and SL used by Medallists and Finalists are highlighted to permit a visual comparison to their peers, as well as visual annotations highlighting the SR-SL combinations used by the gold medallist for the first and final lap of each race distance.

## Results

### Correlational analysis between stroke metrics and speed

Table 1 shows Spearman correlation coefficients between SR and SL with swimming speed across all freestyle distances for men and women. In general, SR was more positively associated with swimming speed as race distance increases, while the relationship between SL and swimming speed weakened over longer distances.

**Table 1:** Spearman correlation coefficients with *p*-values between both stroke rate and stroke length and swimming speed for each race distance.

Race distance [m]	Sex	Stroke-rate	*P* value	Stroke-length	*P* value
50	Men	−0.19	*0* *.* *01*	0.57	*<0* *.* *001*
	Women	0.08	*0* *.* *33*	0.51	*<0* *.* *001*
100	Men	0.34	*<0* *.* *001*	0.28	*<0* *.* *001*
	Women	0.32	*<0* *.* *001*	0.25	*<0* *.* *001*
200	Men	0.30	*<0* *.* *001*	0.36	*<0* *.* *001*
	Women	0.28	*<0* *.* *001*	0.23	*<0* *.* *001*
400	Men	0.25	*<0* *.* *001*	0.26	*<0* *.* *001*
	Women	0.44	*<0* *.* *001*	−0.06	*0* *.* *08*
1500 (M)/800 (W)	Men	0.37	*<0* *.* *001*	0.07	*0* *.* *01*
	Women	0.45	*<0* *.* *001*	−0.08	*0* *.* *01*

M: men; W: women. Yellow indicates negative correlations, green positive; colour intensity reflects correlation strength. Red highlight denote non-significant values.

In the 50 m event, SL had the strongest correlation with speed (men: *ρ* = 0.57; women: *ρ* = 0.50), whereas SR showed trivial or non-significant associations. By the 100 m, both SR and SL showed small-to-moderate correlations with speed in both sexes. In middle-distance races (200 m and 400 m), SR maintained small-to-moderate positive correlations with speed, particularly among women in the 400 m (*ρ* = 0.44), while SL showed variable associations. For long-distance events (1,500 m men, 800 m women), SR was moderately correlated with speed (men: *ρ* = 0.37; women: *ρ* = 0.45), while SL had negligible associations.

### Visualization of SR–SL combinations using 2D KDE plots

**Figure 1:**
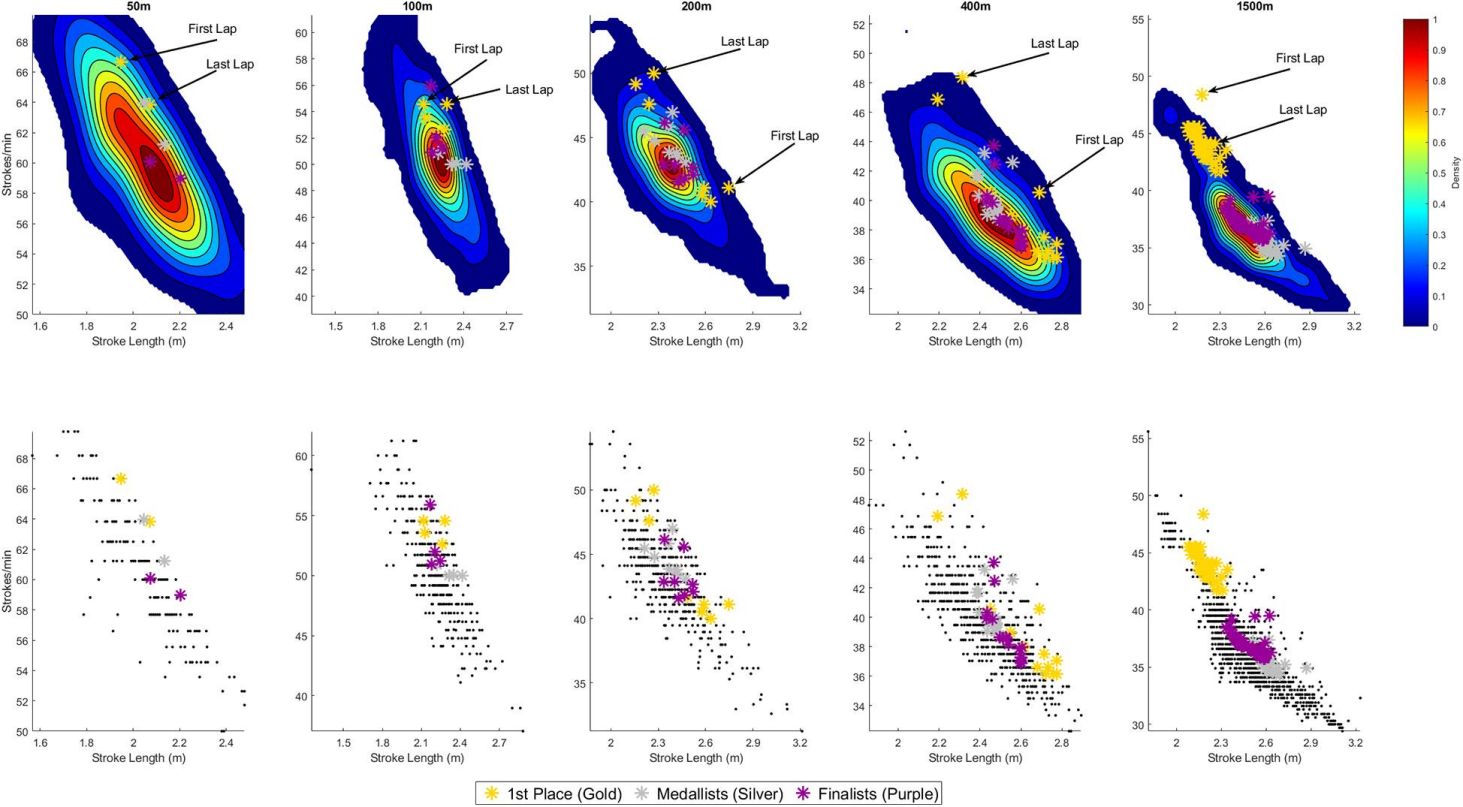
Stroke rate—stroke length combinations for each lap across all race distances for **men**. The **top row** presents continuous two-dimensional (2D) Kernel Density Estimation (KDE) plots, providing a smoothed heatmap showing where data points are most densely concentrated. Each plot represents the probability density of stroke rate and stroke length combinations—areas with warmer colours (e.g., red/yellow) indicate regions where more observations occur, while cooler colours (e.g., blue) reflect less frequent combinations. Visual annotations highlight the SR-SL combinations used by the gold medallist for the first and final lap of each race distance. The **bottom row** illustrates scatter plots which show the raw, unsmoothed data points for each distance. Each black dot represents an individual observation (i.e., one swimmer's stroke rate and stroke length for a single lap). For all plots, the gold stars indicate the stroke rate and stroke length combinations of the gold medallist for every lap, silver stars represent the average stroke rate and stroke length combinations for every lap for medallists (i.e., 2nd and 3rd place) and purple stars represent the average stroke rate and stroke length combinations for every lap for finalists (i.e., 4th to 8th place), providing a visual reference point relative to the rest of the field.

**Figure 2:**
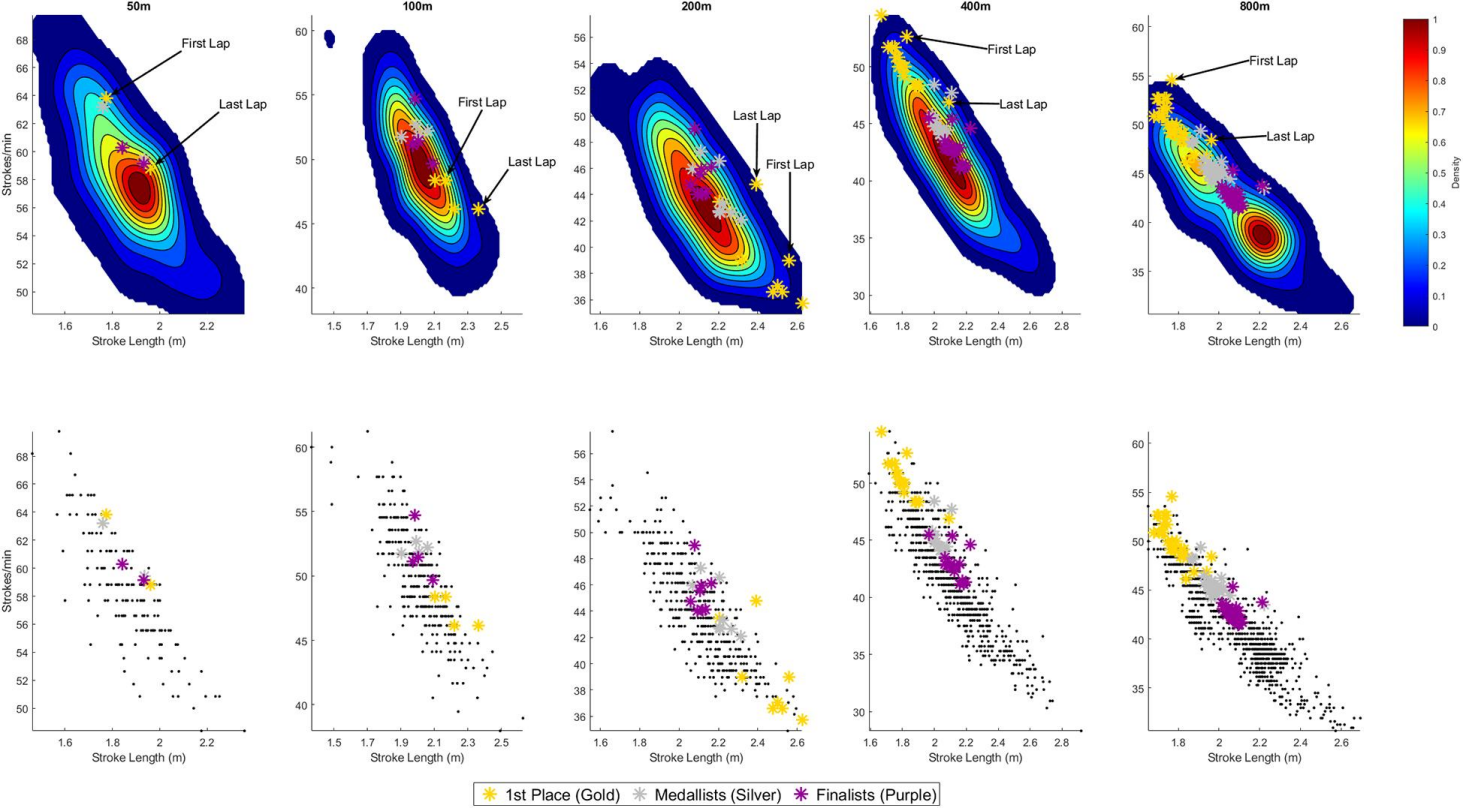
Stroke rate—stroke length combinations for each lap across all race distances for **women**. The **top row** presents continuous two-dimensional (2D) Kernel Density Estimation (KDE) plots, providing a smoothed heatmap showing where data points are most densely concentrated. Each plot represents the probability density of Stroke rate and stroke length combinations—areas with warmer colours (e.g., red/yellow) indicate regions where more observations occur, while cooler colours (e.g., blue) reflect less frequent combinations. Visual annotations highlight the SR-SL combinations used by the gold medallist for the first and final lap of each race distance. The **bottom row** illustrates scatter plots which show the raw, unsmoothed data points for each distance. Each black dot represents an individual observation (i.e., one swimmer's Stroke rate and Stroke length for a single lap). For all plots, the gold stars indicate the stroke rate and stroke length combinations of the gold medallist for every lap, silver stars represent the average stroke rate and stroke length combinations for every lap for medallists (i.e., 2nd and 3rd place) and purple stars represent the average stroke rate and stroke length combinations for every lap for finalists (i.e., 4th to 8th place), providing a visual reference point relative to the rest of the field.

Figures 1, 2 display the distribution of SR and SL combinations across all freestyle events for male and female swimmers, respectively. The upper row shows the smoothed density distributions using 2D KDE, while the lower row presents the corresponding raw data as scatter plots. Across all distances and both sexes, a clear inverse relationship between SR and SL is evident, with most swimmers clustering along a downward-sloping diagonal—swimmers achieving higher SRs tend to exhibit shorter SLs, and vice versa. This trend is especially pronounced in the longer distances where a broader spread of SR–SL strategies are observed.

### Men

#### Sprint-events

In 50 m freestyle, the distribution of SR and SL combinations is tightly clustered. Most swimmers adopt high SR (∼58–62 strokes·min^−^¹) and relatively short SL (∼2.00–2.20 m), as indicated by the dark red regions in the KDE plot. The gold medallist (gold-coloured stars) follows this general pattern but stands out with a higher SR (∼64–67 strokes·min^−^¹), positioning them at the upper end of the cluster. Other Medallists (silver-coloured stars) and Finalists (purple-coloured stars) also occupy positions near the core density zone, but with slightly greater-than-average SR. The two stars for each group of swimmers (winner, medallists, finalists) are not clearly aligned, which indicates changes in the swimming technique or race tactics from the first to the last lap. Specifically, among the gold medallist in the 50 m, SR decreased from 67 to 64 strokes·min^−^¹ from the first to last lap, while SL increased from 1.9 to 2.1 m.

In the 100 m freestyle, the density peak shifts towards lower SR (∼48–52 strokes·min^−^¹) and longer SL (∼2.2–2.3 m), reflecting a trade-off between speed and efficiency. Notably, the gold medallist adopts a higher SR than the densest region, without a corresponding decrease in SL. This is visually evident from the gold stars (gold medallist) appearing above—but not to the right of—the main density area and shows the quality of the winner to achieve higher SR than average and at the same time maintain the SL. Other Medallists and Finalists show a broader distribution than in the 50 m, with generally lower SR and higher SL than the gold medallist, suggesting increased individual variation in pacing and technique over this longer sprint distance. For the gold medallist, SR remained constant from first to last lap (55 strokes·min^−^¹), while SL declined from 2.3 to 2.1 m.

#### Middle-distance events

In the 200 m and 400 m freestyle events, the SR–SL combinations are more widely distributed, with the KDE plots revealing broader, more diffuse density zones compared to the sprint events. The peak densities tend to centre around moderate SR (∼38–45 strokes·min^−^¹) and longer SL (∼2.2–2.6 m). The gold medallists in both events adopted a varied approach. Their data points consistently lie on the periphery of the main density regions—never in the densest zones—indicating that world-class performers leverage stroke strategies that differ from the norm. This includes lap-to-lap variations: some laps show lower-than-average SR paired with above-average SL, while others reveal the opposite—higher SR with reduced SL. Medallists and Finalists in these events are often near the main density peaks, with some laps showing above-average SR. Specifically, for both the 200 m and 400 m events, the gold medallists began with a SR of 41 strokes·min^−^¹ and a SL of 2.7 m. By the final lap, SR increased to 50 strokes·min^−^¹ in the 200 m and 48 strokes·min^−^¹ in the 400 m, while SL decreased to 2.3 m in both cases.

#### Distance-event

In 1,500 m freestyle, the KDE plot reveals the largest distribution of SR–SL combinations across all events, reflecting a wider range of individual strategies. The general trend shifts further toward lower SR (∼35–38 strokes·min^−^¹) and longer SL (∼2.3–2.6 m). Interestingly, the gold medallist showed a clear deviation from this trend. Their technical characteristics showed a clearly higher SR and shorter SL than the population average. The other medallists only showed a trend that deviated from the average and tended to opt for a greater SL and a lower SR. The Finalists were typically close to the main density peak. For the gold medallist, SR declined modestly from 48 to 44 strokes·min^−^¹ between the first and last lap, while SL increased from 2.2 to 2.3 m.

### Women

#### Sprint-events

In 50 m freestyle, the KDE reveals a tight clustering of swimmers around high SRs (∼56–58 strokes·min^−^¹) and short stroke lengths (∼1.8–2.0 m), highlighting a strong emphasis on turnover speed over distance per stroke. The gold medallist's SR–SL combination lies near the peak density region, with a trend towards an above-average SR. This aligns with the men's 50 m event, where the gold medallist also operated at a slightly above-average SR and outside the densest cluster. The other Medallists and Finalists showed a similar trend, with a pattern for above-average SR, indicated by the stars displayed above the main density peak. For the gold medallist, SR decreased from 64 to 59 strokes·min^−^¹ from the first to last lap, while SL increased from 1.8 to 2.0 m.

In 100 m freestyle, the density shifts modestly toward slightly lower SR (∼47–52 strokes·min^−^¹) and somewhat longer SL (∼2.0–2.2 m). The gold medallist again occupies a position close to the central KDE region but exhibits slightly longer-than-norm SL (∼2.1–2.4 m). In comparison, for the other Medallists and Finalists, a higher SR without corresponding increase in SL was observed. For the gold medallist, SR decreased slightly from 48 to 46 strokes·min^−^¹ between the first and last lap, while SL increased from 2.2 to 2.4 m.

#### Middle-distance events

In 200 m freestyle, there is a continued trend toward lower SR (∼41–45 strokes·min^−^¹) and longer SL (∼2.1–2.2 m), reflecting the increasing importance of efficiency. The gold medallist's SR-SL combination lies in the bottom right of the KDE peak, indicating a comparatively lower SR and longer SL, compared to the field. Other Medallists and Finalists typically were positioned close to the main density peak. For the gold medallist, SR increased from 39 to 45 strokes·min^−^¹ between the first and last lap, while SL declined from 2.6 to 2.4 m.

In 400 m freestyle, the KDE spread is noticeably broader, indicating more variation in pacing and technique. Interestingly, the gold medallist's SR–SL combination lies at the upper-left periphery of the density peak (relatively high SR and shorter SL), which contrasts markedly with the pattern observed in the 200 m event. Also, this is opposite to the pattern exhibited by the men's gold medallist, where the SR-SL combinations tended to shift to the bottom right of the KDE plot. Other Medallists and Finalists had SR-SL combinations positioned closer to the main density peak but with a trend for slightly above-average SR. For the gold medallist, SR declined from 53 to 47 strokes·min^−^¹ across the race, while SL increased from 1.8 to 2.1 m.

#### Distance-event

The 800 m freestyle shows a distinctly bimodal distribution in the KDE plot, with one cluster centred around moderate SR (∼39–40 strokes·min^−^¹) and longer SL (∼2.2 m), and a second around higher SR (∼45–46 strokes·min^−^¹) paired with shorter SL (∼1.9 m). Notably, all medallists fell within this second cluster, indicating a strategy favouring higher SR over SL, which was the same as for the 400 m freestyle. In contrast, other Finalists were positioned between the two clusters, adopting a combination of moderately elevated SR and reduced SL—higher SR than the field average, but not to the extent seen in the medallists. For the gold medallist, SR declined from 55 to 48 strokes·min^−^¹ between the first and last lap, while SL increased from 1.8 to 2.0 m.

## Discussion

This study highlights patterns of SR and SL adopted by elite-level swimmers at each freestyle race distance. In particular, we aimed to identify the most common SR–SL combinations among finalists and medallists and examine how these combinations vary by sex and freestyle race distance. A secondary purpose was to develop practical visualisations of these SR-SL combinations that enables the comparison of an individual swimmer's stroke mechanics to those of world-class benchmarks, facilitating technique refinement and strategic planning based on real-world competition data.

The main findings of this study were:

1. Stroke–speed relationships are highly event-specific:
  • In sprint events (100 m and especially 50 m), SL had the greatest correlation with swimming speed, with SR having little or even negative association—which might suggest the importance of maximizing propulsion per stroke at high speed.
  • In middle-distance events (200 m and 400 m), both SR and SL had moderate correlations with swimming speed, which might reflect a need for a balanced strategy combining tempo and efficiency.
  • In long-distance events (800 m/1,500 m), swimming speed was more strongly associated with SR than SL, possibly indicating a reliance on high turnover to maintain pace.
2. Gold medallists often diverged from the field's central tendencies, as visualised using 2D kernel density estimation (KDE). These heat maps revealed that top performers frequently positioned themselves in less densely populated regions of the SR–SL space, suggesting that success at the elite level may involve unique, individualised stroke strategies.
3. Sex-specific differences in SR–SL strategies were observed across distances, with clear variation in the point at which successful swimmers transitioned from favouring above-average SL and below-average SR to the prioritization of higher SR and relatively lower SL (1,500 m for men; 400 m for women).

### Sprint events (50 m and 100 m)

In sprint events, particularly the 50 m freestyle, SL emerged as the dominant determinant of swim speed, while SR was weakly associated or even negatively correlated (notably for men). These results are in line with previous studies that suggest how different level swimmers are able to reach similar SR; however, performance differences appear to stem from their ability to effectively apply force in the water (27). Swimmers who are able to maximise propulsion and cover greater distances per stroke at high velocities demonstrate superior performance (5). This finding aligns with existing biomechanical theory, that SR is an important contributor to sprint speed, however overexciting the pattern may lead to deficient swimming technique and reduced stroke efficiency if not paired with effective SL (10, 28). However, the KDE heat maps provide additional nuance. In the men's 50 m and 100 m events, gold medallists tended to adopt higher SR without a corresponding decrease in SL, as evidenced by density zones shifting vertically (increased SR) but remaining horizontally stable (consistent SL). This finding complements the correlation analysis, as it shows how top male sprinters sustain high SR without compromising SL—reflecting not just efficiency but a greater capacity to generate propulsion over short durations. Their elevated technical ability and power output allow them to select a SR-SL combination to optimise speed and cover the same distance in less time. Rather than maximizing one variable at the expense of the other, top sprinters appear to operate in a performance zone where both SR and SL are pushed toward their upper limits. Moreover, it underscores the pattern that gold medal winners adopt movement pattern that deviate from the norm of their peer group to achieve their extraordinary performances. Additionally, it shows the complementary value of KDE visualisations, which can reveal patterns that simple correlation metrics might obscure. As such, 2D KDE plots can serve as a valuable as complimentary tool to correlation analysis in the context of performance analysis in swimming.

In contrast, the women's 100 m winner favoured a longer SL strategy over high SR, while the 50 m women's winner leaned toward a high SR approach. Although interpretation here is limited due to fewer data points in that distance category, the observed differences may be attributed to the distinct physiological demands of each event (29). Sustaining the very high SR observed during the 50 m races over 100 m may not be feasible for swimmers, leading to a reduction in SR to levels that are generally sustainable and could explain the absence of a strong association. Consequently, at this stage, those swimmers who can achieve greater SL achieve greater velocities. These findings suggest that even in sprint events, success is not about simply maximising SR. Instead, elite swimmers appear to optimise the interplay between SR and SL, with strategies varying by sex, event, and possibly individual strengths such as start performance or anaerobic power.

### Middle-distance events (200 m and 400 m)

In 200 m and 400 m events, both SR and SL were moderately and positively associated with swim speed (except for SL women's 400 m), indicating a more balanced contribution of stroke tempo and stroke distance to performance. This balance reflects the hybrid nature of middle-distance swimming, which demands both aerobic efficiency and high-intensity output. KDE plots revealed more variability among gold medallists in these events, which may relate to the swimmer's distance specialization. As some 200 m swimmers also compete in the 100 m, others may be oriented towards longer distances (30). Stroke pattern in the middle-distance events may therefore be affected by the swimmer's 2nd race distance and explains the larger variability among the gold medallists.

In the women's 400 m, the gold medallist used a relatively high SR and lower SL, suggesting a strategy more akin to long-distance pacing. In contrast, the women's 200 m winner relied on a longer SL, demonstrating a more glide-oriented strategy. For men, gold medallists displayed a lap-to-lap variation: some laps featured lower-than-average SR and longer SL, while others—likely during the final sprint—showed higher SR and shorter SL. This pattern reflects tactical modulation of stroke mechanics, which is characteristic of experienced middle-distance swimmers. While few studies have reported within-race stroke variability during 400 m events, earlier research showed that higher-level swimmers demonstrated greater variability between rounds (31). This suggests an ability to strategically modulate effort stroke mechanics to optimise peak performance.

### Long-Distance events (1,500 m/800 m)

In the longest events, swim speed was more strongly correlated with SR than with SL. This indicates that faster swimmers can sustain significantly higher SR, even if it results in some compromise to SL. The 2D KDE visualisations indicate that both male and female gold medallists in the 1,500 m and 800 m races adopted SR–SL profiles characterised by higher SR and comparatively lower SL than the majority of their peers, consistent with the notion that increased turnover can support sustained propulsion, as observed in open water swimming (32). This finding challenges the traditional coaching emphasis on maximizing SL as the primary determinant of speed in long-distance events, as embodied in metrics such as the stroke index, and underscores the importance of considering SR in training design. However, these findings also suggest that elite distance swimmers possess the physiological capacity to maintain high SR over long durations without compromising performance. In this context, higher SR appears to be not merely an economical pacing strategy, but a sustainable and effective approach made possible by superior conditioning and technique.

Experimental work further supports this perspective. Franken et al. (33) demonstrated that increasing SR at submaximal intensities improved time to exhaustion and modulated oxygen uptake kinetics, suggesting that maintaining a higher turnover can be an effective endurance strategy. Similarly, a flume-based study on submaximal freestyle (34) showed that reducing SR increased oxygen uptake, heart rate, and perceived exertion, whereas increasing SR had minimal effect on these parameters. Together, these studies indicate that elite swimmers may benefit from sustaining higher SR in long-distance events, even at the expense of some SL, because this strategy can maintain speed without substantially increasing physiological strain. Our observations, in which long-distance gold medallists adopted SR–SL combinations emphasizing higher SR, align with these findings and highlight the potential value of SR-focused training for endurance swimming.

### Sex-specific differences in SR–SL strategies

The 2D KDE plots show that male swimmers typically adopt higher SR than females across all race distances. This difference is particularly pronounced in sprint events such as the 50 m, where male finalists cluster above 65 strokes·min^−^¹, compared to female finalists who tend to cluster around 57–59 strokes·min^−^¹. These patterns help explain how female gold medallists often achieve competitive velocities by employing longer-than-average SL combined with moderate SR, positioning themselves near or slightly beyond the KDE density peaks. In contrast, male champions—particularly in sprint events—frequently exceed the high-SR boundary of the KDE distributions, leveraging greater upper-body power and anaerobic capacity to sustain these elevated rates (35, 36). In distance events, both sexes tend to deviate from the broader field patterns, adopting a strategy characterised by relatively higher SR and lower SL, possibly as a response to overcome fatigue towards the end of the race and efficiency demands over longer durations (32).

Notably, a point of transition in the SR-SL relationship over distance differs between sexes. Among male swimmers, a clear pivot occurs between the 400 m and 1,500 m events, shifting from a preference for longer SL and lower SR to a higher SR–lower SL approach. For women, this point of transition appears to happen earlier—between the 200 m and 400 m—suggesting that the biomechanical and energetic demands of long-distance freestyle may assert their influence at shorter race distances in women. This difference may reflect underlying physiological characteristics; for example, females endure a greater percentage of their aerobic capacity than males (37), which could support sustained high SR earlier across race distances. In sprint events, maximizing SL at high SR is a key differentiator, but in longer races, the ability to maintain elevated SR over time becomes the more decisive factor for performance.

Additionally, differences in the Olympic program at the time of the data collection for the present study (up to the 2020 Olympics women only competed in 800 m and men in the 1,500 m freestyle) may have motivated women to spread their physiological capacity from the 800 m to the 400 m in order to increase their medal chances resulting in similar stroke mechanics for both events. This finding highlights the need to tailor stroke strategies not only to race distance and sex-specific performance characteristics, but also to the prevailing competition program, a reason that may change training and race tactics for the sprint events with the 2028 Olympics, where also the 50 m butterfly, backstroke and breaststroke events will be held.

## Limitations

Although several correlations between SR, SL, and swimming speed were statistically significant, the magnitude of these correlations were generally small to moderate. This highlights the multifactorial nature of swimming performance, where SR and SL, while critical, represent only part of a broader system of performance determinants. Other influential factors include start and turn efficiency—especially relevant in short-course races (38, 39)—along with the effectiveness of underwater dolphin kicking (40), race tactics and pacing strategies (41), and an athlete's physiological capacities such as maximal oxygen uptake and lactate tolerance (42). Additionally, neuromuscular control and fatigue resistance play important roles, particularly in sustaining stroke mechanics under competitive pressure (32).

The variability observed in SR–SL strategies both across and within events further illustrates the individuality inherent in elite-level performance. What constitutes an optimal strategy for one swimmer may not apply to another, as many top performers appear to deviate from the central tendencies of the field. This individualised approach to stroke mechanics is effectively visualised in the 2D KDE heat maps, which reveal how athletes often adopt unique solutions rather than conforming to a single ideal model, especially considering that he best combination of SR and SL varies between swimmers, but it could also vary within swimmers (1). It should also be noted that anthropometric characteristics—such as arm span, arm span-to-height ratio, and limb segment lengths—can strongly influence stroke mechanics and the resulting SR–SL profile. The inclusion of such measures in future studies, particularly when examining case studies of Olympic gold medallists, could provide important context for interpreting why certain athletes adopt specific stroke strategies.

## Practical implications

The findings from this study highlight the value of 2D KDE plots as a visual performance analysis tool, allowing coaches to compare a swimmer's SR–SL profile with both the field's central tendencies and the profiles of the most successful competitors. By integrating race analysis data with these visualisations, coaches can develop training interventions that optimise the combination of SR and SL, rather than targeting either in isolation.

Based on the present observations:

Sprint events (50–100 m): Because gold medallists tended to achieve higher velocities through comparatively longer SL with little or even negative association between SR and speed, it may be worthwhile to prioritise training that maximises propulsion per stroke. This could involve strength and power development and technical refinement to maintain SL under race-pace conditions.

Middle-distance events (200–400 m): Because medal-winning swimmers showed moderate correlations between both SR and SL with speed, suggesting a balanced approach, it may be beneficial to design training that develops both turnover and efficiency in parallel. Mixed-pace sets and technique-focused work at varying SRs could support this balance.

Long-distance events (800–1,500 m): Because top performers often relied more heavily on high SR than SL to maintain pace, training might prioritise sustaining elevated SR without loss of efficiency, with an emphasis on rhythm, breathing control, and fatigue resistance.

Sex-specific strategy shifts: Because male and female gold medallists differed in the event distance at which they transitioned from above-average SL to above-average SR (1,500 m for men, 400 m for women), it may be worthwhile to monitor each swimmer's natural transition point and tailor training to optimise it in line with these tendencies.

The KDE visualisations further indicate that gold medallists often operated in sparsely populated SR–SL regions, suggesting that success may depend on developing and refining individualised stroke strategies rather than strictly following the event average. Coaches could therefore use this approach to identify advantageous deviations from the norm and track how these evolve over the training cycle.

## Conclusion

This study reinforces the concept that optimal stroke mechanics are event-specific in elite swimming. By combining traditional correlation analysis with advanced visualisation techniques like 2D KDE, we gain a clearer picture of how top performers optimise the balance between SR and SL. Across race distances, SR–SL strategies evolve in distinct ways: shorter events are characterised by high SR and relatively lower SL, while longer distances tend to involve a shift toward more balanced or even SL-dominant approaches—although this pattern differs between sexes. Correlation analyses further revealed that SR becomes more strongly associated with swim speed as race distance increases, while the influence of SL weakens over longer distances.

Despite these general trends, the 2D KDE visualisations demonstrate that medallists do not always conform to them. For instance, although SL showed a strong positive correlation with swim speed in the 50 m event for both men and women, the gold medallists adopted a strategy featuring above-average SR, rather than relying on longer SL. This suggests that while group-level relationships provide useful context, they do not fully capture the performance strategies that distinguish podium finishers from the broader field. Notably, these swimmers appear able to reach and sustain exceptionally high SR without incurring the typical losses in SL—likely due to superior technique, strength, and neuromuscular control. Such findings highlight that excellence in swimming is not defined by uniformity, but by the skilled execution of individualised race strategies.

Importantly, this study provides coaches and sport scientists with a data-informed reference framework for performance evaluation and athlete development. The 2D KDE heat maps offer a practical and intuitive tool for comparing an individual swimmer's stroke mechanics to world-class benchmarks. This enables more targeted technique refinement and strategic planning grounded in real-world championship data, helping to bridge the gap between biomechanical analysis and applied coaching practice.

## Data Availability

The raw data supporting the conclusions of this article will be made available by the authors, without undue reservation.
